# Effects of Long and Short Carboxylated or Aminated Multiwalled Carbon Nanotubes on Blood Coagulation

**DOI:** 10.1371/journal.pone.0038995

**Published:** 2012-07-10

**Authors:** Jie Meng, Xuelian Cheng, Jian Liu, Weiqi Zhang, Xiaojin Li, Hua Kong, Haiyan Xu

**Affiliations:** Institute of Basic Medical Sciences, Chinese Academy of Medical Sciences & Peking Union Medical College, Beijing, People’s Republic of China; Emory University/Georgia Insititute of Technology, United States of America

## Abstract

In this work the effects of four different multiwalled carbon nanotubes (MWCNTs), including long carboxylated (L-COOH), short carboxylated (S-COOH), long aminated (L-NH_2_) and short aminated (S-NH_2_) ones, on the integrity of red blood cells, coagulation kinetics and activation of platelets were investigated with human whole blood. We found that the four MWCNTs induced different degrees of red blood cell damage as well as a mild level of platelet activation (10–25%). L-COOH and L-NH_2_ induced a higher level of platelet activation than S-COOH and S-NH_2_ respectively; meanwhile L-NH_2_ caused marked reductions in platelet viability. The presence of the four MWCNTs led to earlier fibrin formation, L-NH_2_ increased the clots hardness significantly, while L-COOH and S-NH_2_ made the clots become softer. It was concluded that the four MWCNTs affected blood coagulation process and the clots mechanical properties; they also altered the integrity of the red blood cells and the viability of the platelets, as well as induced platelets activation. The effects of MWCNTs depended on the size and chemistry of the nanotubes and the type of cells they contacted.

## Introduction

Carbon nanotubes have been explored in novel delivery systems for drugs [1]–[3] or DNA/RNA [4]–[7], as imaging contrast agents [8], and as detection devices for capturing tumor cells from blood [9], which implies an interaction between carbon nanotubes and blood elements. Hence, the potential clinical use of carbon nanotubes will be critically dependent on their toxicity to blood cells. The interactions between carbon nanotubes and blood may lead to blood coagulation and thrombosis. Although carbon nanotubes showed improvement effects on composite materials blood compatibility when they were used as fillers or in fabric forms [10]–[14], several groups have reported that single or multiwalled carbon nanotubes in particulate status could induce platelets activation and aggregation [15]–[20]; in which Simak group indicated the mechanism of carbon nanotubes-induced platelets activation, showing that pristine multiwalled carbon nanotubes activated platelets by causing depletion of intracellular Ca^2+^ stores [19] as well as inducing extracellular Ca^2+^ influx [20]. Nevertheless, the carbon nanotubes tested by different groups are obtained from various commercial sources and different in structure, length and surface chemistry, such as multiwalled [15], [17]–[20] or single walled [15], [17], [19]), pristine [15], [18]–[20], carboxylated and aminated [18]), longer as several micrometers [19] or shorter less than one micrometer [16], which make very hard to compare their blood compatibility regarding to be applied in blood contact environments. In addition, the details of interactions between carbon nanotubes and blood still remain largely unexplored. For example, the damage of red blood cells and hemoglobin release is also one important process that is involved in blood coagulation. However, the influences of carbon nanotubes on red blood cells and thrombus mechanical properties have been rarely reported [21]. One another issue is the coagulation kinetics that can provide crucial cues to imply the alternation of coagulation function in pathological conditions, which has been hardly reported in carbon nanotube-blood interactions. The aim of this study was to investigate the role of different length of MWCNTs with carboxylated or aminated surface in blood coagulation using well-characterized MWCNTs of the same source and compare these findings to effects reported in the literature. In particular, variations of red blood cells morphology and thrombus mechanical properties induced by the different MWCNTs were addressed.

## Results and Discussion

### Characterization of the Four MWCNTs

In the present study, two carboxylated multiwalled carbon nanotubes (L-COOH and S-COOH) were prepared through a combined oxidation procedure of concentrated acids and probe sonication from pristine long and short multiwalled carbon nanotubes (MWCNTs) respectively. The resulting average lengths were 926 nm and 223 nm for L-COOH and S-COOH respectively. Aminated MWCNTs were obtained through amidation of L-COOH and S-COOH with 1, 6-diaminohexane, the average lengths were 945 nm and 266 nm for L-NH_2_ and S-NH_2_ respectively. XPS analysis indicated that the percentage of carboxylic carbon atoms was 4.66% and 3.62% respectively. The L-NH_2_ and S-NH_2_ contained 2.19% and 3.16% of amidated carbon atoms respectively. FTIR spectrum of L-COOH showed that the characteristic absorption of carboxylic group at 1720 cm^−1^ substantially decreased in the L-NH_2_ spectrum, while an absorption peak at 1630 cm^−1^ appeared; S-COOH and S-NH_2_ exhibited similar absorption spectrum to L-COOH and L-NH_2_ respectively. The characterization information above mentioned was given by **Figure S1** and **Figure S2**.

### The Four MWCNTs Influenced the Morphological Variation and Membrane Integrity of Red Blood Cells

**Figure 1:**
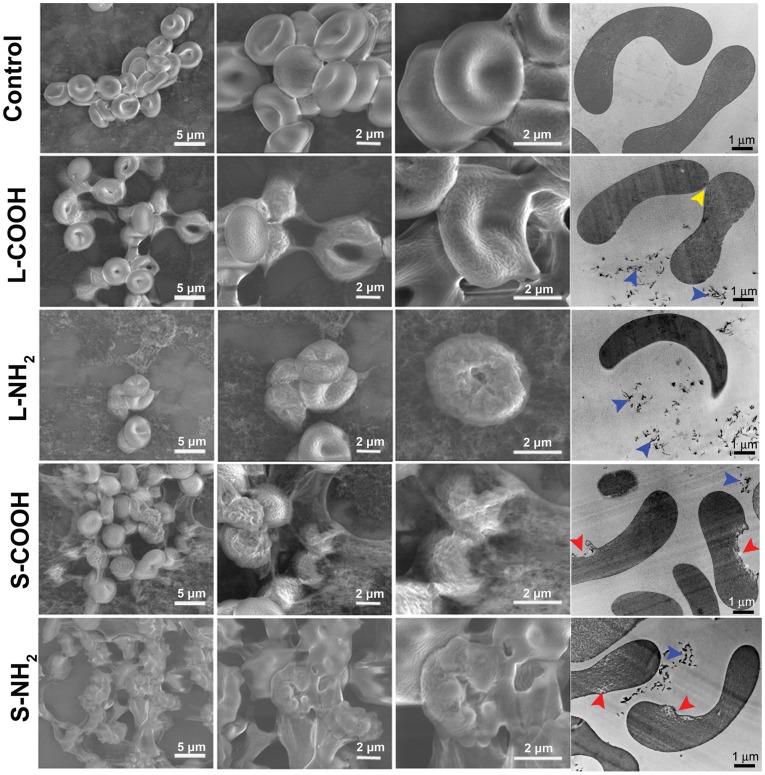
ESEM observation of membrane morphology for RBCs exposed to L-COOH, L-NH_2_, S-COOH and S-NH_2_ of 0.16 mg/ml at 37°C for 2 h.

Red blood cells (RBCs) are the most common cells in blood and a principal carrier of oxygen to body tissues, containing hemoglobin inside their membrane. We used Environmental Scanning Electron Microscopy (ESEM) and Transmission Electron Microscopy (TEM) to explore the interaction between the four MWCNTs and RBCs, in particular to localize the MWCNTs and integrity of the RBCs. It is interesting to see that the MWCNTs exhibited influence upon the RBCs in size dependent manner (Figure 1). It could be noticed from the TEM images that there were MWCNTs around the RBCs (blue arrow head), however, no MWCNTs inside the RBCs. This might owing to the low engulf capacity of RBCs. In addition, few MWCNTs were seen adhered on the RBCs surface, suggesting that affinity of MWCNTs to the RBCs was low. As for MWCNTs-induced morphological changes, in general the long MWCNTs mainly induced RBCs shrunk, distortion and aggregated (yellow arrow head), while the short MWCNTs caused RBCs membrane damage (red arrow head). We would addressed that in sample preparation RBCs in L-NH_2_ group were significantly reduced than the other groups, which suggested L-NH_2_ was more hemolytic than the others. Further comparison showed that aminated MWCNTs had stronger RBCs toxicity than carboxylated ones when the MWCNTs had similar size.

Images captured from ESEM provided further details of the RBCs morphological changes. Normal RBCs typically displayed a biconcave-disk liked appearance (control). After incubation with L-COOH, the cells membrane became “sticky” and adhered to each other. Additionally, many cells lost the smooth surface, clumped together, and took on a crumpled, slightly shrunken, or even crushed appearance. After interacting with L-NH_2_, RBCs mainly displayed a shrunken appearance, and their membranes became quite rough. Particularly it was difficult to find as many cells in L-NH_2_ treated samples compared to the other three treatments, implying that many RBCs had been damaged after incubated with L-NH_2_. After incubated with S-COOH or S-NH_2_, clumping and distortion became more severe; we observed more crushed cells and larger RBC aggregations than observed with L-COOH and L-NH_2_. In particular, after exposure to S-NH_2_, few intact RBCs could be observed. Schar *et al* have shown that carboxylated SWCNTs loosely bound to RBC membranes resulted in significant reduction in RBC viability in the mouse blood, from 70% to 14% [21]. In the present study, significant changes in RBC membrane integrity and cells number were observed in the human whole blood exposed to the four MWCNTs. These results suggested that the four MWCNTs had cytotoxic effects on RBCs. They appeared to cause cell distortion and membrane disruption. For damage level assessment, the two aminated MWCNTs caused more severe distortion of RBCs than the two carboxylated MWCNTs when they have similar length; while shorter MWCNTs exhibited stronger influence upon RBCs integrity than the longer ones when they have similar surface chemistry.

**Figure 2:**
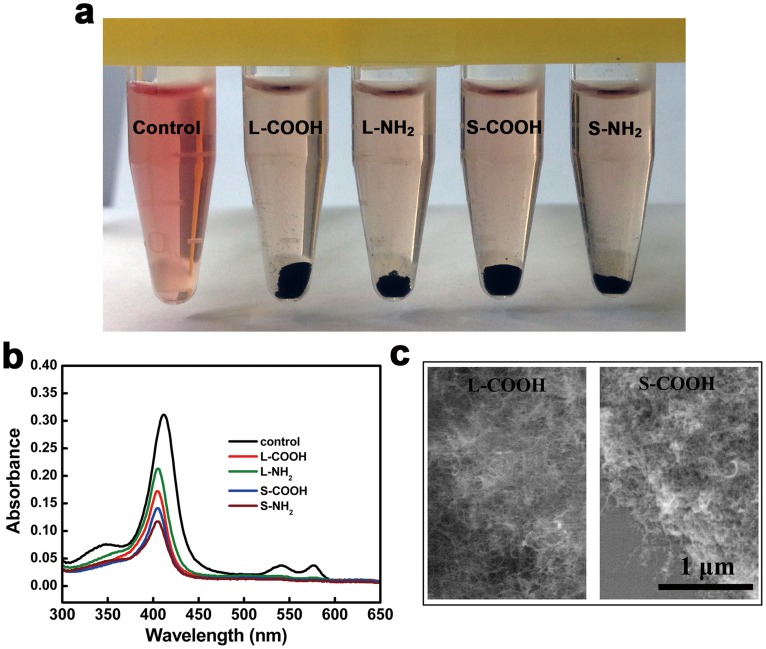
Adsorption of Hb released from RBCs to the MWCNTs. (a) Supernatants obtained by ultra high speed centrifugation of the diluted Hb solution exposed or nonexposed with the MWCNTs. (b) Absorption spectra of the diluted Hb solution exposed or nonexposed with the MWCNTs. (c) ESEM images of the MWCNTs after incubated in the diluted Hb solution, showing strong adsorption of Hb to the MWCNTs.

When RBCs are damaged, Hb is released and the extent of hemolysis is usually assessed by optical spectroscopy based on the characteristic absorption of Hb. However, carbon nanotubes can nonspecifically, strongly adsorb to various proteins such as lysosozyme [22], serum albumin [23], plasma proteins [24], and the complement component [25]–[26], which may pull the proteins out of the tested solution and is likely interfere with optical absorption results [27]. Considering the protein adsorption characteristics to carbon nanotubes, we prepared a dilute Hb solution by inducing RBC lysis in water and examined whether the Hb was adsorbed to the four MWCNTs. The four MWCNTs were added into the diluted Hb solution and incubated for 2 h at 37°C, and then removed by ultra high speed centrifugation. The supernatant of the intact diluted Hb solution (as positive control) had a light red color, indicating Hb had remained in the supernatant, while the Hb solutions that had been exposed to the four MWCNTs showed no red color, implying that most of the Hb was adsorbed to the four MWCNTs and depleted from the supernatants (**Figure 2a**). The supernatants were further subjected to optical spectroscopy to determine the variation of Hb concentration by the OD intensity at 400 nm. The absorbance spectra showed that the peak intensity at 400 nm decreased with MWCNTs exposure (**Figure 2b**) in the following order S-NH_2_ (70%) > S-COOH (67%) > L-COOH (61%) > L-NH_2_ (53%) compared to the control Hb, implying the adsorption degree of Hb to the four MWCNTs. ESEM examination of the MWCNTs sediment showed large aggregations of the MWCNTs due to Hb adsorption (**Figure 2c** **)**. These results also implied that hemolysis induced by carbon nanotubes was not available to be examined quantitatively in the conventional hemolytic activity assay.

### The Four MWCNTs Caused Platelet Activation and Lost Viability

**Figure 3:**
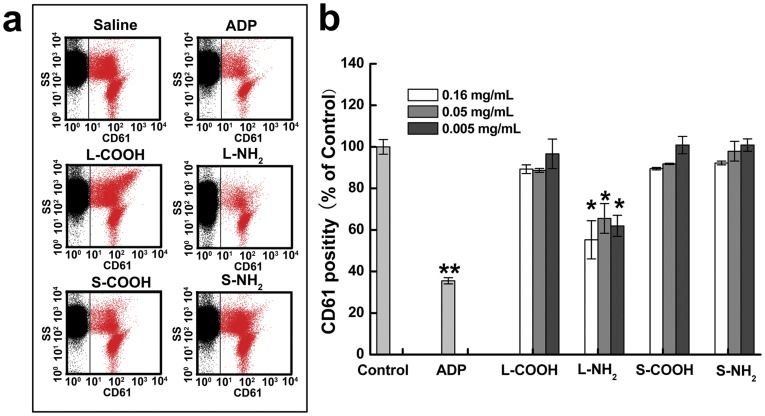
Effects of the MWCNTs on the platelets viability. (a) Flow cytometric analysis plots of the anticoagulant blood exposed with the MWCNTs at 0.16 mg/ml. The red dots represent viable platelets that express CD61. (b) Percentage of the CD61^+^ platelets in the blood. *: significantly different from the negative control (*p<0.05, **p<0.01). Values represent mean ± SD.

Platelets play a vital role in blood coagulation. Both resting and activated platelets express the membrane protein, CD61, which is an indicator of platelet viability. **Figure 3** showed representative flow cytometry graphs of CD61-positive platelets in the whole blood after incubation with the four MWCNTs. Among the four MWCNTs, L-NH_2_ caused the largest reduction in the CD61-positive platelets; this indicated that L-NH_2_ was cytotoxic to platelets, and the other three MWCNTs did not affect platelet viability significantly. Burker *et al.* reported that significant decrease in circulating platelets was observed at 3 h in mice injected with carboxylated MWCNTs in pluronic F127, while the influence of aminated MWCNTs dispersed in PEG or in pluronic F127 was indistinguishable from control [18]. The difference might be associated with the addition of surfactants PEG and F127. Additionally, different experimental models might also result in different results. In current experiment platelets were counted in the human whole blood using anti-CD61 antibody, instead, in the literature experiment, platelets were counted in the platelet rich plasma of mice under microscope. Besides, different size distribution and hydrophilicity of MWCNTs used in the different research groups was likely another reason that might lead to different effects on platelets viability.

**Figure 4:**
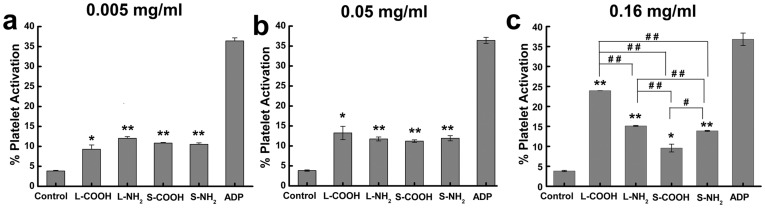
Comparison of platelets activation induced by the four MWCNTs at different concentrations. (a) 0.005 mg/ml; (b) 0.05 mg/ml; (c) 0.16 mg/ml. *: significantly different from the negative control (*p<0.05, **p<0.01); #: significant difference between the indicated MWCNTs (#*p*<0.05, # *p*<0.01). Values represent mean ± SD.

For this study, CD62p was selected to indicate activated platelets. As shown in **Figure 4** **,** a very low number of platelets expressed CD62p in the negative control (saline), indicating majority of the platelets remained inactivated. The four MWCNTs induced moderate platelet activation, and ADP caused the high platelet activation compared to the negative control. A general tendency was observed that the four MWCNTs induced a level about 10% of platelet activation at the concentration of 0.005 mg/ml and 0.05 mg/ml in reference to the negative control, however, there were no significant differences observed in the platelet activation induced by the four MWCNTs. At the highest concentration of 0.16 mg/ml, L-COOH induced higher level of platelet activation than S-COOH; similarly, L-NH_2_ induced higher expression of CD62p than S-NH_2_. Among the four MWCNTs, S-COOH induced the lowest platelet activation. In general, long MWCNTs induced higher levels of platelet activation than short MWCNTs when they had similar surface chemistries. Several groups have reported interactions between carbon nanotubes and platelets. Simak *et al* assessed the effect of different kinds of carbon nanotubes on platelet aggregation with platelet rich plasma (PRP), including two commercial available MWCNTs different in diameter and length distribution. Comparing the reported data, the MWCNTs with higher aspect ratio exhibited increasing tendency of platelet aggregation activity [19]. These results supported our observation that longer carbon nanotubes activated platelets more effectively than shorter carbon nanotubes.

### The Four MWCNTs Altered Clot Hardness and Coagulation Kinetics

Both RBC hemolysis and platelet activation initiate the coagulation process and lead to the formation of thrombi. Thromboelastography (TEG) can measure abnormalities in the coagulation cascade in blood by detecting the elastic properties of blood clots as they form. In TEG blood coagulation and clot strength are graphically represented over time in a characteristic cigar-shaped figure, in which four indexes of the TEG trace were used to probe the dynamics of blood coagulation and the strength of the final clot. The *R* was the time for initial fibrin formation; the *k* represented the time from the beginning of clot formation until 20 mm of amplitude. The *α* was the slope between R and k, which reflected clot strengthening. The *MA* was the maximum amplitude, which represented the maximum strength of the clot; this depended on the number and function of platelets and their interaction with fibrin [28]. A representative TEG graph was given by **Figure S3**, in which the four indexes were indicated.

**Figure 5:**
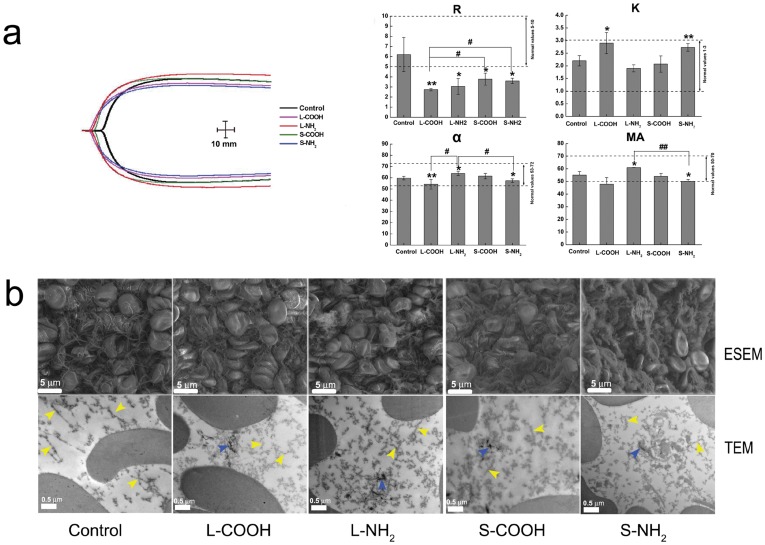
Comparison of coagulation kinetics of the blood after exposure to the MWCNTs. (a) representative thromboelastography traces for untreated blood (black trace) and blood treated with the MWCNTs; the unit for x- and y- axis is mm. (b) Variations induced by the MWCNTs in the four key coagulation parameters. Dashed lines indicate the normal limits.

The TEG analysis showed that the four MWCNTs caused changes in *R*, *k*, *α*, and *MA* values in the cigar-shaped figure to different degrees (**Figure 5a**). Compared to control, *R* was significantly decreased by the four MWCNTs. This indicated that the initial coagulation process occurred earlier than normal when blood samples were incubated with the four MWCNTs. The *k* and *α* value of the four MWCNTs were changed to different degrees, L-COOH and S-NH_2_ induced lower *α* values and higher *k* values than untreated control samples, but the values were within the control reference range. This implied that these MWCNTs induced a tendency for slower clot formation and resulted in a softer clot compared to controls. The *MA* values induced by the four MWCNTs were within the normal reference range, except that of L-COOH. The L-COOH induced a significantly lower *MA* than the other three NWCNTs and the control; this also implied that L-COOH induced a softer clot. Taken together, the four MWCNTs affected the coagulation function of the blood to different extents; they significantly accelerated fibrin formation, but slowed down clot formation, and reduced clot hardness. Murugesan *et al* showed that pristine MWCNTs did not affected TEG clotting kinetics [29]. Those pristine MWCNTs had an average diameter of 40 nm and length of 10 µm and could not be dispersed in solution. The discrepancy with our results strongly suggested that coagulation behavior was largely dependent on the physical and chemical characteristics of MWCNTs.

We have also conducted TEM and ESEM with the blood clots formed in exposure to the MWCNTs (Figure 5b). Typical ESEM images showed that the four MWCNTs induced structure changes of fibrin network in the clots to different extends. The fibrin fibers in the control clots were continuous with smooth surface, and the network structure was clear. In contrast, the fibrin fibers in the clots formed in exposure to the four MWCNTs were rough and short, and did not form clear network structure. Further comparison showed that short and amidated MWCNTs induced more severe changes of fibrin fibers themselves as well as the network they formed. Observations of TEM showed that the MWCNTs existed in the networks formed by the randomly entangled fibrin fibers. It could be seen that the fibrin network in the clot formed in exposure to L-NH_2_ was much more dense in reference to control, while that in the exposure to S-NH_2_ was more loose than control. These observations were consistent with the results of TEG. Hence it is rational to consider that the MWCNTs disturbed the fibrin fibers to form normal network. The abnormal fibrin fibers and networks reduced the clots elasticity and resulted in the increase of clot hardness.

### Conclusion

Taken together, the effects of MWCNTs on blood cells largely depended on the size and surface chemistry as well as the cell type. Despite the MWCNTs were rarely engulfed by the RBCs, they induced morphological changes of the red blood cells as well as membrane destruction to different degrees. They activated platelets (10–25%), among the four MWCNTs, L-NH_2_ caused a marked reduction in platelet viability. These interactions affected the clotting formation kinetics and led to earlier fibrin formation. The L-NH_2_ increased the clots hardness significantly, while S-NH_2_ made the clots become softer. Our studies indicate that cautions should be taken when using carbon nanotubes in blood contact environments.

## Materials and Methods

### Blood Collection

The protocol for this study was reviewed and approved by the Institutional Review Board of Chinese Academy of Medical Sciences & Peking Union Medical College. All the subjects participated in the study after providing informed consent. Native whole blood was collected from consenting informed healthy volunteer donors that had not taken medication for at least 10 days prior to donation. The blood was collected into vacutainer tubes containing 3% of sodium citrate solution to obtain anticoagulant blood samples for uses in blood exposure assays; the volume ratio of blood to sodium citrate solution was 9∶1.

### Preparation of Surface Modified MWCNTs

Pristine MWCNTs that were long (50 µm) or short (0.5–2 µm) were purchased from Chengdu Organic Chemicals Co. Ltd. (Chengdu, China), the product code was TNM2 and TNM5 respectively. To prepare carboxylated MWCNTs (L-COOH or S-COOH), the two pristine MWCNTs were treated with an oxidation/sonication procedure, as described previously [30]. In brief, 100 mg of pristine long or short MWCNTs was added to 100 ml of a mixture of concentrated H_2_SO_4_ and HNO_3_ (3∶1 by volume) and dispersed with probe sonication at 750 W for 80 seconds (working mode: working 2 seconds following 2 seconds stop), the diameter of the probe was 10 mm. The mixture was then diluted with an aqueous solution of NaOH followed by vacuum filtration through a polycarbonate filter membrane of 2 mm pore and washing with a large amount of pure water to reach neutralization. The filtrates were dried completely in a vacuum oven at 50°C to obtain dried L-COOH or S-COOH.

To obtain aminated MWCNTs (L-NH_2_ and S-NH_2_), 10 mg of the dried L-COOH or S-COOH was dispersed in 10 ml of pure water to obtain a solution of 1 mg/ml by the aid of probe sonication (Sonication time: 60 seconds, working model: working 3 seconds following 3 seconds stop; working power was 600 W). Next, 10 mg of 1-ethyl-3-(3-dimetylaminopropyl) carbodiimide hydrochloride (EDC, Sigma) and 13 mg N-hydroxysulfosuccinimide sodium salt (Sigma) was added to the solution and allowed to react for 15 min. After that, 6.7 mg of 1, 6-diaminohexane dissolved in 1 ml of tetrahydrofuran was added drop by drop and stirred at room temperature for 2 h to have the carboxylic groups’ amidated with amine groups. The reaction products were transferred to a membrane bag and dialyzed against pure water to remove unreacted molecules (cutoff molecular weight of 12000 Da). After that, the solution in the bag was conducted vacuum filtration through a polycarbonate filter membrane of 2 mm pore and washing with pure water. The filtrates were dried completely in a vacuum oven at 50°C to obtain dried L-NH_2_ or S- NH_2_.

### Characterization of the Surface Modified MWCNTs

The prepared L-COOH, S-COOH, L-NH_2_, and S-NH_2_ were characterized by scanning electron microscopy (SEM, Hitachi S-5200) to examine their morphology, X-ray photon spectroscopy (XPS, Japan JEOL Scientific JPS-9010TR) and Fourier transform infrared spectroscopy (FTIR, Necolet NEXUS 670) to analyze their surface chemistry.

### Preparation of MWCNTs Suspension for Blood Exposure Assays

The powder samples of the four MWCNTs were sterilized by autoclaving, then dispersed in pure water to obtain a stock solution of 1 mg/ml by the aid of probe sonication (Sonication time: 60 seconds, working model: working 3 seconds following 3 seconds stop; working power was 600 W). Priot to conducting the blood exposure experiments, the stock solution was diluted by pure water to obtain two diluted solutions of 0.31 mg/ml and 0.031 mg/ml; next the solution of 1.0 mg/ml, 0.31 mg/ml and 0.031 mg/ml was mixed with 4.5% NaCl aqueous solution (4∶1 by volume) to obtain three solutions of 0.8 mg/ml, 0.25 mg/ml, and 0.025 mg/ml for uses in the follwing blood exposure assays.

### Assessment of Red Blood Cell Morphology

The anticoagulant blood of 0.5 ml was added to 19.5 ml of saline to obtain diluted blood. The solution of 0.8 mg/ml of the four MWCNTs was quickly added and mixed with the diluted blood (1:4 by volume) followed by incubation at 37°C for 2 h. After that, the incubated mixtures were centrifuged at 750×g for 5 min to obtain cell sediments.

For ESEM observation, the cell sediments were resuspended in PBS, the suspension was deposited on clean glass slides and fixed with 1% glutaraldehyde in 0.1 M phosphate buffer (pH 7.4). After that the deposited cells were washed with the same buffer, dehydrated with a gradient of ethanol solutions (50, 70, 95 and 100%), and dried at critical-point. The resulting samples were subjected to environmental scanning electron microscopy (ESEM, Quanta 200 FEG, FEI Co.).

For TEM observation, the cell sediments were fixed in 1% glutaradehyde in 0.1 M phosphate buffer (pH 7.4) overnight, washed by the same buffer and post-fixed with 1% (w/v) osmium tetroxide for 30 min. After washed, the samples were dehydrated through graded ethanol and then embedded in Epon-812. Ultrathin sections were cut, double stained with uranyl acetate and lead citrate and placed on a copper meshwork. The sections were observed on a transmission electron microscopy (TEM, JEM-1010).

### Absorption of Hemoglobin to Carbon Nanotubes

The anticoagulant blood of 0.5 ml was mixed violently with 19.5 ml of pure water to lyse the red blood cells (RBCs). The mixture was centrifuged at 12000 rpm/min for 20 min, the supernatant was hemoglobin (Hb) solution. The solution of 0.8 mg/ml of the four MWCNTs was quickly added to the Hb solution and incubated at 37°C for 2 h. After that, the mixture was centrifuged at 12000 rpm/min for 20 min; supernatants were aspirated and subjected to ultraviolet-visible wavelength spectroscopy.

### Thromboelastography Assay for Evaluating Blood Coagulation

The solution of 0.8 mg/ml for the four MWCNTs was quickly added into the anticoagulant blood (1∶4 by volume), followed by incuabtion for 2 h at 37°C. Thus the final concentration of the MWCNTs in the blood was 0.16 mg/ml. Thromboelastography (TEG) traces were obtained with a Thromboelastograph D according to the manufacturer’s instructions (Hellige GMBH, Germany). Briefly, 340 µL of blood naïve or the MWCNTs exposed blood was placed in TEG-sample cups, and 20 µL of isotonic CaCl_2_ solution (0.2 M) was added for recalcification. The cylindrical cup with the blood was oscillated through an angle of *4°45′* at 37°C. Each rotation cycle of the cup lasted 10 s. A stainless-steel cylindrical piston was suspended from a torsion wire and immersed in the cup. The torque of the cup was transmitted to the piston through the fibrin fibers that gradually formed between the piston and the wall of the cup; the rotation of the piston became increasingly strong as the clot solidified.

### Assessment of Blood Clots by ESEM and TEM Observation

Solutions of the four MWCNTs (0.8 mg/ml) were quickly added into the anticoagulant blood in volume ratio of 1∶4, followed by incubation for 2 h at 37°C. After the incubation, 40 µl of isotonic CaCl_2_ solution (0.2 M) was added into 680 µl of the blood samples to initiate blood coagulation. The blood samples were allowed to coagulation at room temperature for 1 h, and then kept at 4°C overnight to make the blood have enough time to form clots. Centrifugation was carried out at 100×g for 10 min to remove the supernatants and obtain the clot samples.

For ESEM observation, the clots were fixed with 1% glutaradehyde and dehydrated with a gradient of ethanol solutions, followed by critical-point drying. The resulting samples were subjected to Environmental Scanning Electron Microscopy (ESEM Quanta 200 FEG, FEI Co.). For TEM observation, the clots were treated with the same protocol for red blood cells.

### Platelet Activation Analyzed by Flow Cytometry

The solution of 0.8 mg/ml, 0.31 mg/ml and 0.031 mg/ml for the four MWCNTs was quickly added into the anticoagulant blood (1∶4 by volume), followed by incuabtion for 1 h at 37°C. Thus the final concentration of the MWCNTs in the blood was 0.16 mg/ml, 0.05 mg/ml, and 0.005 mg/ml respectively. ADP (AMRESCO) of 20 µM as a positive activation control. After incubation, anti-CD61 antibodies conjugated to fluoresce in isothiocyanate (FITC, eBiosciences) and anti-CD62P antibodies conjugated to phycoerythrin (PE, eBiosciences) were added to the samples according to the manufacturer’s instruction. The mouse IgG conjugated to FITC or PE was used as non-specific binding controls. After 20 min of incubation, the blood samples were fixed with 0.5 ml of 1% paraformaldehyde saline solution and analyzed by flow cytometery (Coulter EPICS XL). Data are presented as the mean ± SD.

### Statistical Analysis

Single-factor analysis of variance (ANOVA) was employed to assess the statistical significance of the results. All data are presented as the mean ± SD. P-values <0.05 were considered statistically significant.

## Supporting Information

Figure S1 Chemical analyses of oxidized and amidated carbon nanotubes. (a) C_1s_ spectra of L-COOH and S-COOH. (b) C_1s_ spectra of L-NH_2_ and S-NH_2_. (c) N_1s_ spectra of L-NH_2_ and S-NH_2_. (d) FTIR spectra of L-COOH and L-NH_2_.
(TIF)

Figure S2 SEM images of chemically modified nanotubes. Representative images are shown for (a) L-COOH, (b) L-NH_2_, (c) S-COOH, and (d) S-NH_2_. Inserts show graphs of the length distributions.
(TIF)

Figure S3 A representative TEG graph including the four indexes. The x- and y- axis is mm. The *R* is the time for initial fibrin formation; the *k* represented the time from the beginning of clot formation until 20 mm of amplitude; the *α* is the slope between R and k, which reflected clot strengthening; the maximum amplitude (*MA*) represents the maximum strength of the clot.
(TIF)
