# Supplementary figures and images for: Effects of Long and Short Carboxylated or Aminated Multiwalled Carbon Nanotubes on Blood Coagulation
Source: PLoS One. 2012 Jul 10;7(7):e38995. doi: 10.1371/journal.pone.0038995 (PMC3393720; doi:10.1371/journal.pone.0038995)

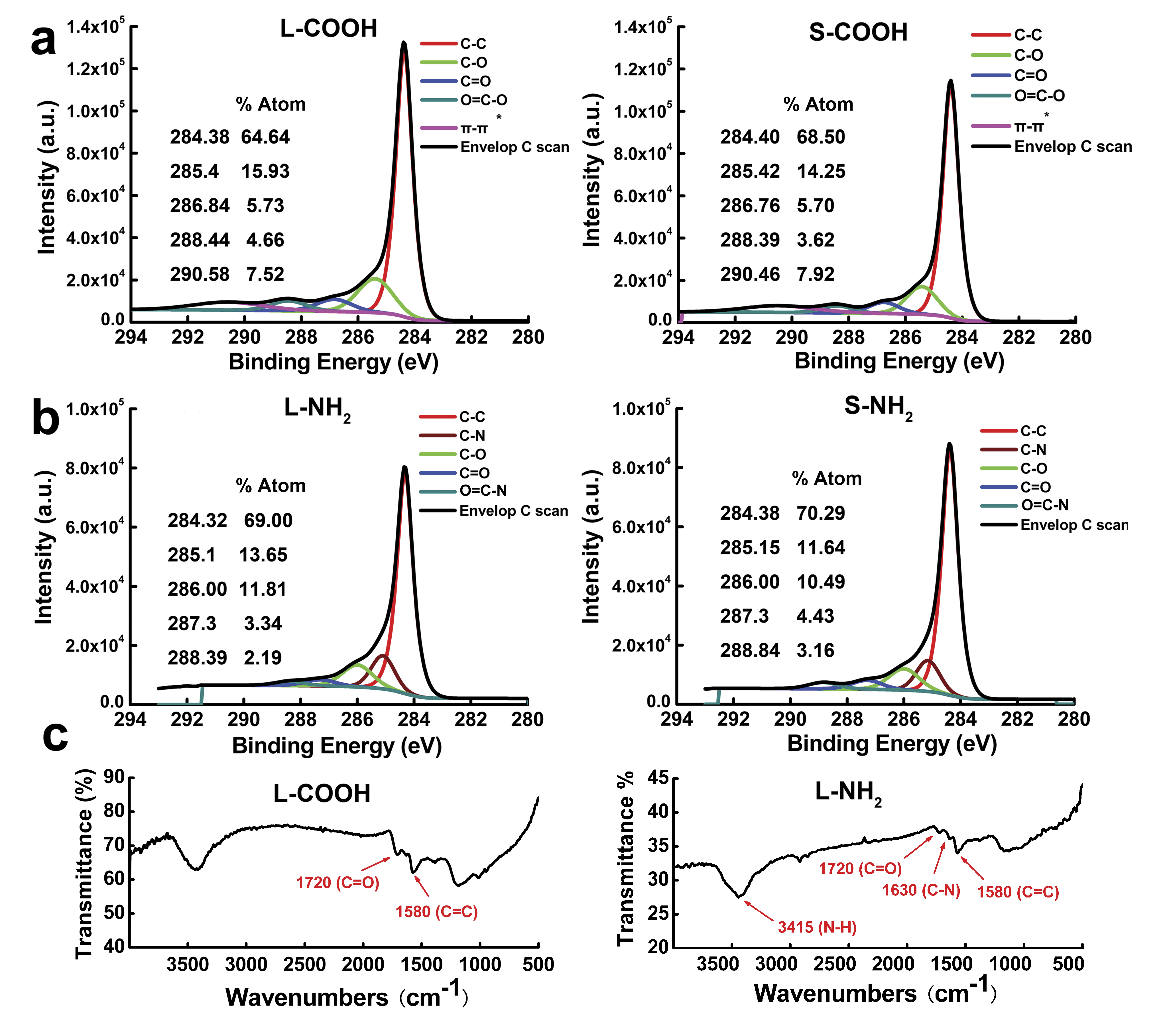

Supplement: Figure S1 — Chemical analyses of oxidized and amidated carbon nanotubes. (a) C1s spectra of L-COOH and S-COOH. (b) C1s spectra of L-NH2 and S-NH2. (c) N1s spectra of L-NH2 and S-NH2. (d) FTIR spectra of L-COOH and L-NH2. (TIF) [file pone.0038995.s001.tif]

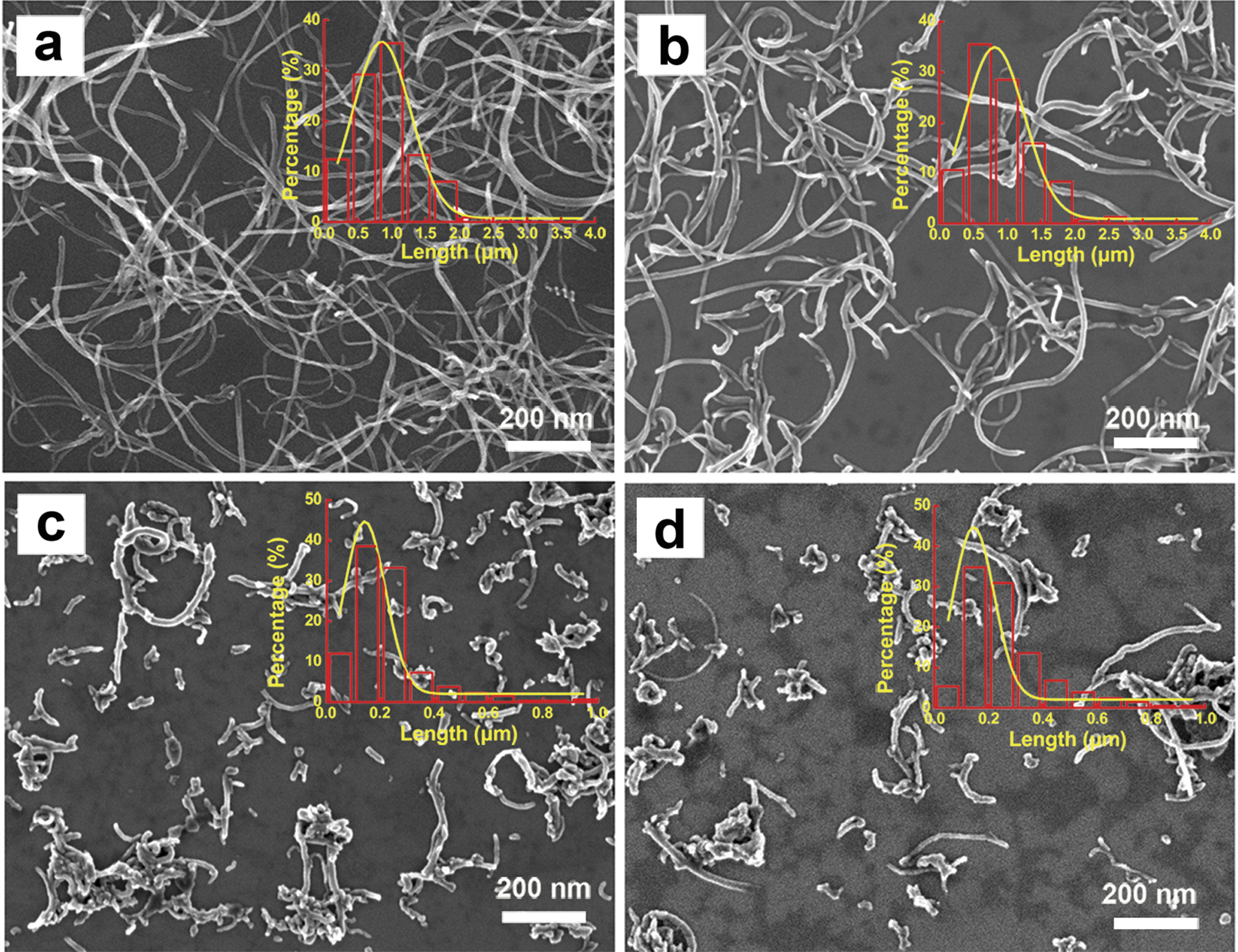

Supplement: Figure S2 — SEM images of chemically modified nanotubes. Representative images are shown for (a) L-COOH, (b) L-NH2, (c) S-COOH, and (d) S-NH2. Inserts show graphs of the length distributions. (TIF) [file pone.0038995.s002.tif]

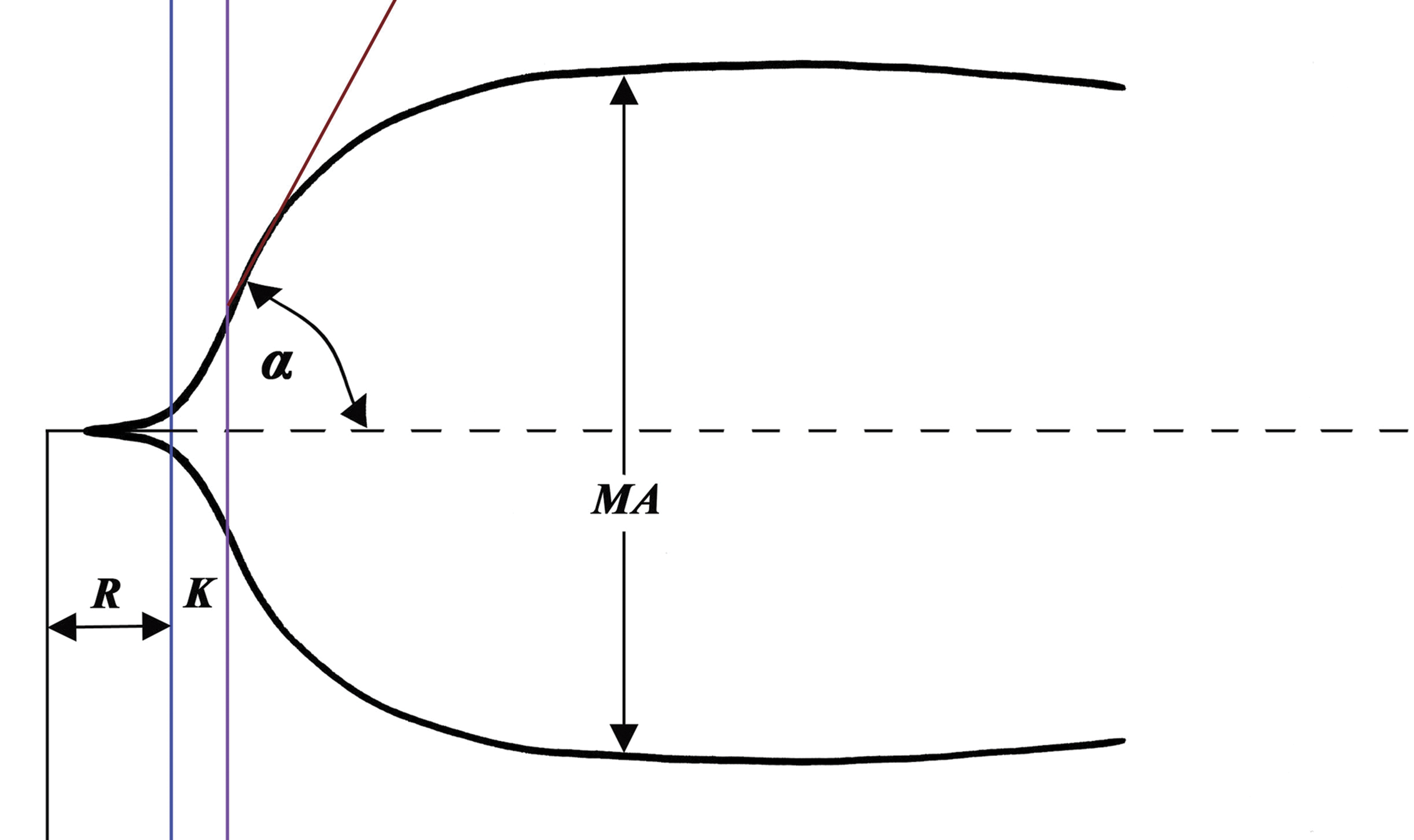

Supplement: Figure S3 — A representative TEG graph including the four indexes. The x- and y- axis is mm. The R is the time for initial fibrin formation; the k represented the time from the beginning of clot formation until 20 mm of amplitude; the α is the slope between R and k, which reflected clot strengthening; the maximum amplitude (MA) represents the maximum strength of the clot. (TIF) [file pone.0038995.s003.tif]
